# Assessment of Trace Metals in Consumer Chickens in Bangladesh

**DOI:** 10.5696/2156-9614-8.20.181208

**Published:** 2018-12-03

**Authors:** Md. Abdul Mottalib, Gulam Zilani, Tarikul Islam Suman, Tusar Ahmed, Shafiqul Islam

**Affiliations:** 1 Institute of Leather Engineering and Technology, University of Dhaka, Dhaka, Bangladesh; 2 TÜV SÜD Bangladesh Private Limited, Dhaka, Bangladesh; 3 Environment and Resource Management, Brandenburg University of Technology Cottbus-Senftenberg, Cottbus, Germany

**Keywords:** toxic metal, poultry, carcinogenic, cadmium, chromium, copper, cobalt, arsenic, Bangladesh

## Abstract

**Background.:**

Bangladesh is a densely populated country with a high demand for non-vegetable protein. Poultry meat is an important source of protein due to its affordability. Trace metals are persistent in the environment and are subject to bioaccumulation in the food chain. Contaminated poultry feed can pose a risk to human health as it biomagnifies and bioconcentrates toxic metals up the food chain, which can enter the human body and cause adverse and toxic health effects.

**Objectives.:**

The aim of the current study was to assess the concentration and distribution of metals such as arsenic (As), cadmium (Cd), cobalt (Co), chromium (Cr), copper (Cu) and nickel (Ni) in different types and parts of poultry in markets in Dhaka, Bangladesh.

**Methods.:**

A total of 15 chickens were collected from five commercial markets of Dhaka city. Three types of poultry were selected, broiler, layer and cock. Altogether, 45 chicken samples were analyzed for toxic metals concentrations using inductively coupled plasma optical emission spectroscopy.

**Results.:**

The concentrations of As, Cd, Co, Cr, Cu and Ni obtained in the broiler, layer and cock chickens were 0.728, 0.232, 0.392; 0.595, 0.245, 0.271; 0.058, 0.016, 0.096; 5.275, 1.562, 22.180; 3.571, 2.269, 4.241, and 0.332, 0.211, 0.433 mg/kg (wet weight), respectively. The results indicate that the concentrations of As, Cr, and Cu in the analyzed samples exceeded the maximum permissible levels, whereas the concentrations of Cd and Ni were within acceptable limits. Metal concentrations decreased in the order of Cr > Cu > As > Cd > Ni > Co. The target hazard quotient and cancer risk values were estimated for each metal due to consumption of the different types of chicken.

**Conclusions.:**

The estimated metal concentrations of As, Cr and Cu were higher than the permissible levels of international standards, indicating a health risk. Groundwater in many parts of the country is contaminated with As and is a probable source of As contamination in poultry. High concentrations of Cr and Cu in chicken may be caused by contaminated poultry feed. In the present study, layer chickens were comparatively less contaminated with metals than other types of chicken. The estimated target hazard quotient and cancer risk values of the analyzed chicken samples were lower than acceptable limits for all individual trace metals in the three types of chicken, indicating no non-carcinogenic and cancer health risk from ingestion of a single trace metal through consumption of these chickens.

**Ethical Approval.:**

This study was approved by the Academic Committee of the Institute of Leather Engineering and Technology, University of Dhaka, Bangladesh.

**Competing Interests.:**

The authors declare no competing financial interests.

## Introduction

Commercial chicken meat is widely consumed in Bangladesh, where it is a major source of animal protein. Chicken meat has high biological value, as it contains essential amino acids required to promote human growth and health. Despite its nutritional benefits, the quality of poultry meat may be affected by the contamination of toxic metals through various anthropogenic activities.^1^ Toxic metal contamination of meat poses a risk to human health, because as metals bioaccumulate up the food chain they can biomagnify and can cause various adverse health effects. Arsenic (As), cadmium (Cd), mercury (Hg) and lead (Pb) pose the greatest risk and have deleterious impacts on human health. Increased concentrations of toxic metals have entered the environment as industrialization continues to advance without pollution prevention measures.^2^ Few studies have investigated toxic metal accumulation and contaminated diets in chickens to assess the potential human consumption risk.^3–6^

Nickel (Ni) increases the risk of lung cancer, cardiovascular disease, neurological deficits, developmental deficits in children, high blood pressure, as well as severely disrupts enzyme activity.^7^ Cadmium is toxic to virtually every system in the body. Cadmium accumulates within the kidney and liver and can cause kidney dysfunction, skeletal damage, prostate cancer, and mutation.^8–10^ Chromium can exist in different oxidation states. As an essential micro element, Cr (III) plays an important role in nutrition. Chromium (III) is a cofactor of insulin, which is involved in glucose, lipid and protein metabolism. However, the hexavalent form of Cr is carcinogenic.^11^

Bangladesh is a densely populated country with a high demand for non-vegetable protein such as poultry meat and eggs, as they are affordable options. Most poultry farms in Bangladesh are maintained with shallow well water which contains relatively more arsenic than deep well water.^12^ Hens raised with arsenic-rich drinking water and feed may accumulate it in their flesh, eggs, and excreta, which can cause contamination in poultry products.^13^ Essential metals such as copper (Cu), zinc (Zn), manganese (Mn) and cobalt (Co) play an important role in the biological system. Cobalt is a core element of vitamin B12, which is essential to human health. Exposure to naturally occurring levels of cobalt in the environment is not harmful, however when exposure is drastically increased, damaging health effects can occur. There are few published studies on the contamination of poultry meat in Bangladesh.^5^,^6^,^14^ As part of our ongoing studies on toxic metal contamination in soil, vegetables, poultry and fish feed, the objective of the current study was to assess the concentration and distribution of As, Cd, Co, Cr, Cu and Ni in different types and parts of poultry.

**Abbreviations**
*THQ*: Target hazard quotient
*TR*: Target cancer risk
*USEPA*: United States Environmental Protection Agency
*WHO*: World Health Organization

## Methods

Chickens that are raised for meat are called broilers. These chickens are typically white and are bred specifically for optimal health and size to produce a quality consumer product. Chickens raised for eggs are called layers and a male chicken is called a cock. Broiler, layer and cock chickens are widely available and comparatively cheap in Bangladesh. Broiler and cock are reared mainly to fulfill the demand of meat in the country, whereas layer chickens provide both meat and eggs. Broiler, layer, and cock chickens consume different amounts of feed due to their different ages and therefore trace metal concentrations in their meat were considered separately. These three types of chicken, broiler, layer and cock, were randomly collected from five commercial markets: Hazaribagh, New Market, Zigatola, Polashi and Kawran bazaar in Dhaka, Bangladesh. These markets were selected to cover a large area of Dhaka. Thousands of consumers buy chickens from these commercial markets every day. Three types of chicken were purchased from each market. A summary of the age and weight of the chickens is shown in Table 1. Three meat samples, breast, head and liver, were collected from each chicken. A total of 45 samples (5 markets × 3 types of chickens × 3 parts = 45 samples) were collected for the present study and analyzed for trace metal contamination.

**Table 1:**
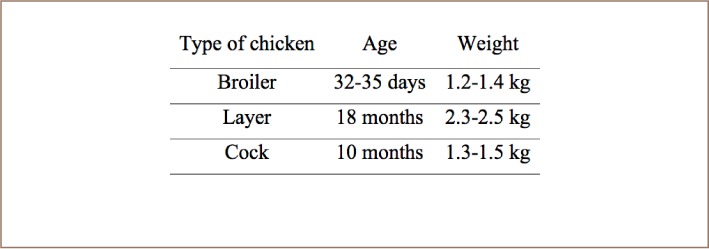
Age and Weight of Different Types of Poultry

### Sample digestion

Once the chickens were slaughtered with a sharp stainless-steel knife, samples (breast, head, liver) were collected in plastic boxes and stored at −18°C and digested as soon as possible. Chicken samples were cut into small, 2 g (wet weight) pieces. Each sample was drawn into a quick-fit conical flask and a mixture of 15 ml concentrated nitric acid and perchloric acid (4:1 vol/vol) was added. The mixture was then digested with a condenser and stirred at 80°C for 2 hours until the solution became colorless.^6^,^15^,^16^ Afterwards, the sample was removed and allowed to cool. The mixture was filtered with Whitman no. 42 filter paper. The conical flask was then rinsed with double distilled water and the filtrate was transferred into a volumetric flask and finally diluted with 25 ml of distilled water. Trace amounts of residue remained on the filter paper. The clear filtrate of each sample was kept in a refrigerator to avoid evaporation.

All knives, glassware (conical flask, funnel, condenser, magnet, and spatula) were rinsed with dilute nitric acid solution and then washed several times with tap water and finally with distilled water. All were dried in an oven at 110°C to remove moisture.

### Sample analysis

All samples were analyzed using inductively coupled plasma-optical emission spectroscopy (Optima-7000 DV, USA). The analytical procedure was checked against a known concentration of the standard reference material purchased from CPAchem, Bulgaria. The percent recovery was between 94–105%, as shown in Table 2.

**Table 2:**
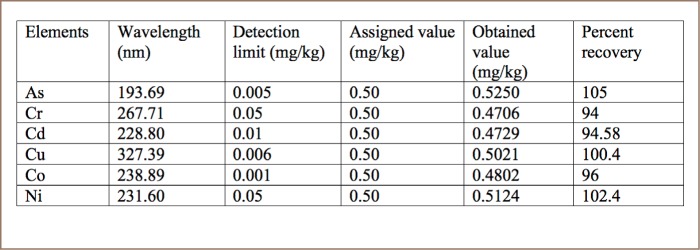
Wavelength, Detection Limit and Percent Recovery of Each Trace Metal

All chemicals (Merck, Germany) used were analytical grade, including standard stock solutions of known concentration for each metal. Blank samples were regularly analyzed after every seven samples. Concentrations were calculated on a wet weight basis. All analyses were replicated three times. The precision and analytical accuracy of the analyses were approved by the analysis of the standard reference material (CPAchem, Bulgaria). Microsoft Excel (Redmond, WA, 2007) was used to assess the concentration of trace metals, including the mean, median, minimum, maximum and standard deviation in the chicken samples. The present study used Equation 1 to calculate metal concentrations (in ppm, wet weight):

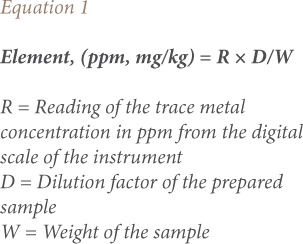



### Health risk estimation

The target hazard quotient (THQ) is an estimate of the risk level (non-carcinogenic) due to pollutant exposure. To estimate the human health risk from contaminated chicken, the THQ was calculated using the United States Environmental Protection Agency (USEPA) Region III Risk-Based Concentration Table.^17^ The equation used for estimating THQ was as follows:

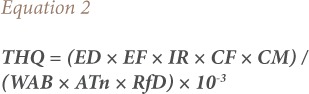
Where THQ is the target hazard quotient, EF is the exposure frequency (365 days/year), ED is the exposure duration (30 years for non-cancer risk as suggested by the USEPA), IR is the ingestion rate of chicken tissue (49.5 g/person/day), CM is the metal concentration in chicken (mg/kg, wet weight), WAB is the average body weight (70 Kg), ATn is the average exposure time for non-carcinogens (EF×ED) (365 days/year) for 30 years (ATn=10,950 days) as used for characterizing non-cancer risk, and RfD is the reference dose of the metal (an estimate of the daily exposure to which the human population may be continuously exposed over a lifetime without an appreciable risk of deleterious effects).^17^,^18^

### Hazard index

The hazard index from THQs is expressed as the sum of the hazard quotients.^17^


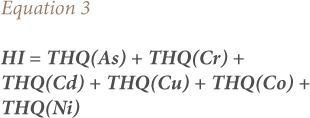


### Target cancer risk

Target cancer risk (TR) was used to indicate carcinogenic risk. The method to estimate TR is also provided in the USEPA Region III Risk-Based Concentration Table.^17^ The model for estimating TR was as follows:

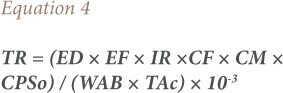
Where TR is the target cancer risk, CM is the metal concentration in chicken (mg/kg, wet weight), IR is the chicken ingestion rate (g/day), CPSo is the carcinogenic potency slope, oral (mg/kg body weight/day), and ATc is the average time for carcinogens (365 days/year for 70 years).^17^ The CPSo values (mg/kg body weight/day) are 1.5, 0.38, 0.7, 0.5, 1.5 and 1.7 for As, Cd, Co, Cr, Cu and Ni, respectively, thus TR values were calculated for intake of these metals.

## Results

The mean concentrations of As, Cd, Co, Cr, Cu and Ni in the breast, head and liver of broiler chickens were 0.633, 0.607, 0.943 mg/kg; 0.243, 0.450, 1.092 mg/kg; 0.061, 0.042, 0.070 mg/kg; 3.976, 10.167, 1.683 mg/kg; 2.422, 4.2, 4.092 mg/kg; and 0.298, 0.299, 0.398 mg/kg, respectively (*Tables 3 and 4*). The results showed the concentration of metals from the three parts of broiler chicken in the following order: Cr > Cu > Cd > As > Ni > Co.

**Table 3:**
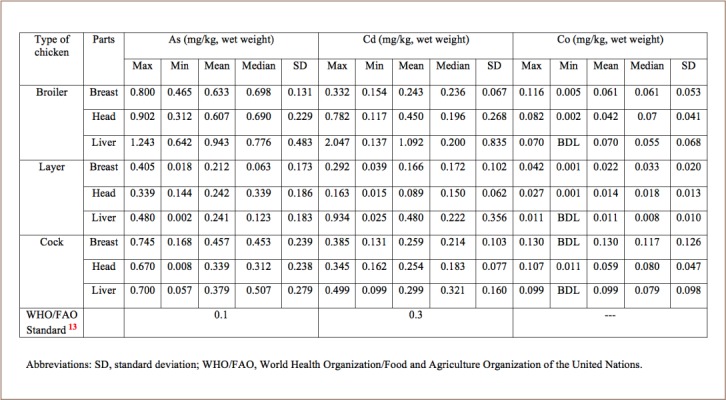
Concentrations of As, Cd, and Co in Chicken Samples Compared with World Health Organization Standards

**Table 4:**
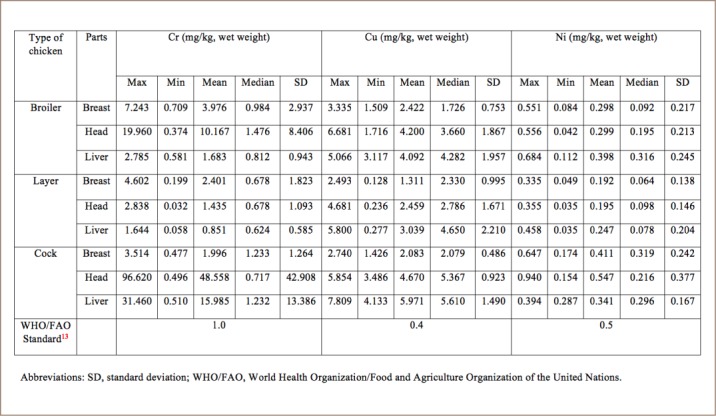
Concentrations of Cr, Cu and Ni in Chicken Samples Compared with World Health Organization Standards

The mean concentrations of As, Cd, Co, Cr, Cu and Ni in the breast, head and liver of layer chickens were 0.212, 0.242, 0.241 mg/kg; 0.166, 0.089, 0.480 mg/kg; 0.022, 0.014, 0.011 mg/kg; 2.401, 1.435, 0.851 mg/kg; 1.311, 2.459, 3.039 mg/kg; and 0.192, 0.195, 0.247 mg/kg, respectively. Lastly, the mean concentrations of As, Cd, Co, Cr, Cu and Ni in the breast, head and liver of cocks were 0.457, 0.339, 0.379 mg/kg; 0.259, 0.253, 0.299 mg/kg; 0.130, 0.059, 0.099 mg/kg; 1.996, 48.558, 15.985 mg/kg; 2.083, 4.67, 5.971 mg/kg; and 0.411, 0.547, 0.341 mg/kg, respectively. The results showed the concentration of metals from the three parts of layer chicken in the following order: Cu > Cr > Cd > As > Ni > Co. The concentration of metals in cock chickens was as follows: Cr > Cu > Ni > As > Cd > Co.

The preliminary results showed that the highest concentrations of Cd, As and Cu were found in broilers, whereas the highest Cr content was detected in cock chickens. The highest concentration of Cr was found in the head samples and lowest in the breast. The highest concentration of Cu was identified in the liver samples (5.80 mg/kg) and lowest in the breast (0.128 mg/kg).

**Figure 1:**
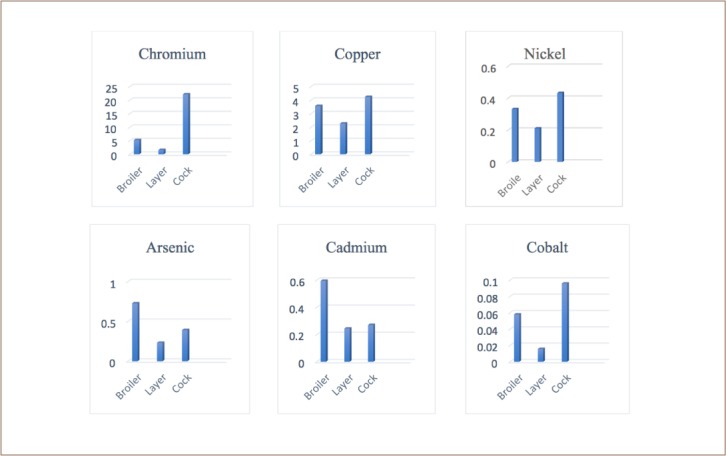
Average metal concentrations (mg/kg, wet weight) in different types of chicken

The average metal concentrations in the different types of chicken are shown in Figure 1. Arsenic ranged from 2.1–7.0 mg/kg. The As content in the samples decreased in the order of broiler > cock > layer. According to the World Health Organization (WHO) standard, the maximum permissible limit of As is 0.1 mg/kg.^13^ All of the concentrations in the studied samples were higher than the maximum permissible limit.

Cadmium concentrations ranged from 0.015–2.047 mg/kg. The highest concentration of Cd (2.047 mg/kg) was found in broiler chicken. The Cd content in the studied samples decreased in the order of broiler > cock > layer. The concentration of Cd found in broiler chickens was higher than the maximum permissible limit, although Cd concentrations were within the permissible limit for both layer and cock chickens. According to the WHO standard, the permissible concentration of Cd is 0.3 mg/kg.^13^

The Co present in the chicken samples ranged from 0.01–0.130 mg/kg. The highest concentration of Co was found in cock chickens (0.130 mg/kg) and decreased in the order of cock > broiler > layer.

Chromium (III) is an essential element at low concentrations, although the hexavalent form of chromium is carcinogenic.^11^ The recommended daily intake for adults is between 0.02 and 0.5 mg/day.^19^ The chromium concentration in the current study ranged from 0.032–96.62 mg/kg. The highest concentration of Cr was found in head samples of cock chickens (96.62 mg/kg), followed by broiler and layer chickens. According to the USEPA, the maximum permissible limit of Cr in chicken meat is 1 mg/kg. The concentration of Cr in cocks was much higher than the maximum permissible limit.

The copper concentrations found in the analyzed samples ranged from 0.128–7.809 mg/kg. The highest concentration of Cu (7.809 mg/kg) was present in cocks. The order of Cu concentrations in the present study was cock > broiler > layer. According to the WHO, the maximum permissible limit of Cu is 0.4 mg/kg.^13^

The tolerance levels of Ni in children and adults are 7 mg/days and 40 mg/days, respectively.^4^ The highest concentration of Ni was found in the head samples of cocks (0.940 mg/kg), the lowest was in liver and head (0.035 mg/kg) of the layer chickens. Comparatively lower concentrations of Ni were detected in layer chickens. All of the values were within the maximum permissible limit of Ni of 0.5 mg/kg.^13^

### Correlation coefficient of metals

The correlation coefficient r measures the strength and direction of a linear relationship between two variables on a scatter plot. The value of r is always between +1 and −1. A correlation value of +1 indicates a perfect positive linear relationship. As one variable increases in value, the other variable also increases. A correlation value of −1 indicates a perfect negative linear relationship. As one variable increases in value, the other variable decreases in value via an exact linear rule. A correlation greater than 0.8 is generally described as strong, whereas a correlation less than 0.5 is generally described as weak.

The Pearson coefficient was used to calculate the correlation between trace metals concentrations and to establish the mutual influence in the bioaccumulation process. Table 5 shows a strong positive correlation between Co-Ni (r^2^=0.950) in broilers. Table 6 shows a strong positive correlation between Cd-Cu (r^2^=0.854) and a significantly positive correlation between Cr-Cu (r^2^=0.508) in layers. Table 7 shows a strong positive correlation between As-Ni (r^2^=0.827), Cd-Cu (r^2^=0.860), Co-Ni (r^2^=0.817), and a significantly positive correlation between Cr-Cu (r2=0.761), As-Co (r^2^=0.594) and Cu-Ni (r^2^= 0.498) in cocks. The strong positive correlation indicates that these trace metals had similar pollution levels and sources. However, each type of chicken shows a significantly high negative correlation with As-Cr.

**Table 5:**
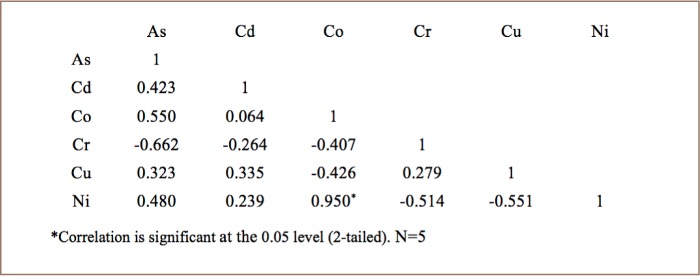
Correlation Matrix Between Metal Concentrations in Broiler Chicken

**Table 6:**
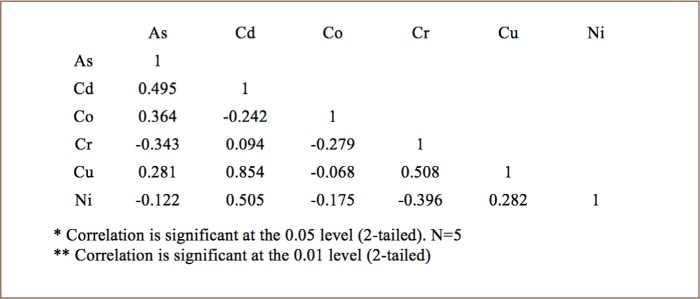
Correlation Matrix Between Metal Concentrations in Layer Chicken

**Table 7:**
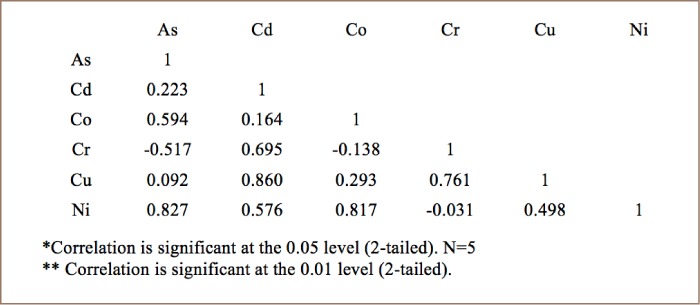
Correlation Matrix Between Metal Concentrations in Cocks

## Discussion

The concentration of As levels recorded in this study were higher than those reported by Akan et al. (0.3 mg/kg).^2^ Elsharawy found As concentrations in chicken samples between the range of 0.36 – 0.85 μg/g.^20^ Several studies have shown that inorganic arsenic can cause lung, bladder, liver, kidney, prostate and skin cancer. Emerging science also shows that inorganic arsenic may harm pregnant women and fetuses. Arsenic has been shown to cross the placenta to the fetus and has been found in breast milk. Chronic exposure to arsenic has been shown to affect child development by lowering IQ scores.

The range of Cd concentration (0.015–2.047 mg/kg) in the current study was substantially lower than the Cd contents (2.67–4.33 mg/kg) found in chicken in Bangladesh in a study by Rahman et al.^5^

Cadmium levels found in the current study were similar to the levels reported in previous studies 0.012–0.227 mg/kg and 0.45–2.23 mg/kg in different tissues of chicken respectively,^21^,^6^ and higher than those reported by Gonzalez-Weller et al. (0.0017 mg/kg),^22^ Bari et al. (0.01 to 0.031 mg/kg),^23^ Skalicka et al. (0.019 to 0.061 mg/kg)^24^ and Orisakwe et al. (0.234 mg/kg)^25^ and lower than 0.059 to 9.36 mg/kg.^26^

The concentrations of Co levels recorded in the present study were similar to the levels reported in previous studies (0.095 mg/kg)^27^ and (0.007–0.030 mg/kg),^28^ and lower than those reported by Abdel-Salam et al. (0.2 mg/kg)^29^ and higher than 0.04 mg/kg.30 Cobalt concentrations between 0.062–7.08 mg/kg in chicken have been reported by Kar et al.^31^

The chromium concentrations recorded in this study were higher than those reported by Iwegbue et al. (0.01–3.43 mg/kg).^4^ The mean concentration of Cr (0.032–96.62 mg/kg) was higher than reported in China (0.430 mg/kg, Wang et al. and 0.158 mg/kg, Yuanan et al.) and in northern Nigeria (2.55 mg/kg, Orisakwe et al. 2017).^25^,^28^,^32^ In general, the concentration of total chromium in meat ranged from 0.01 to 1.3 mg/kg.

The Cu concentrations detected in the present study were very similar to the results obtained by Alturiqi and Albedair (2.31 to 7.79 mg/kg), and higher than those recorded by Mahmoud Elsharawy (0.15–1.16 μg/g), Orisakwe et al. (1.012 mg/kg) and Menezes et al. (0.65–0.84).^20^,^25^,^33^,^34^ Copper concentrations in meat increase with the age of the chicken and depend on the concentration of Cu in feed.^31^ Although Cu is an essential nutritional element, high intakes can cause health problems such as liver and kidney damage (Agency for Toxic Substances and Disease Registry, 2004).^35^ Copper is an important constituent in a number of different enzymes.

Nickel plays a pivotal role in human health, however, high levels of Ni may result in serious respiratory distress and cancer. The mean Ni content in chicken in the present study (0.035–0.940 mg/kg) was lower than the values reported in Dareta, Northern Nigeria (1.012 mg/kg).^25^ The maximum concentration of Ni obtained in the current study was higher than in previous studies reported by Wang et al. (76.5 μg/kg), Zahurul et al. (0.491 mg/kg) and Yuanan Hu et al.^27^,^32^,^36^

### Health risk estimation

The risk associated with the carcinogenic effects of a target metal is expressed as the probability of contracting cancer over a lifetime of 70 years. The THQ estimated for the individual metals via consumption of different types of chicken is shown in Table 8. If the THQ value is less than 1, the risk of non-carcinogenic toxic effects is assumed to be low.^17^ The THQ values of the analyzed chicken samples were less than 1 for all individual trace metals in the three types of chicken. The results indicate that ingestion of a single trace metal through consumption of these chickens should have no non-carcinogenic health risk. Target cancer risk values exceeding 1×10^−4^ are regarded as intolerable, a risk value lower than 1×10^−6^ is regarded as safe, and risk values between 1×10^−4^ and 1×10^−6^ are considered satisfactory.^37^ An acceptable cancer risk value is 1×10^−6^ according to the USEPA, 1996.^17^ The results of current study revealed that the TR values of the analyzed samples for all trace metals were within the acceptable limit, indicating no cancer health risk from the consumption of these chickens.

**Table 8:**
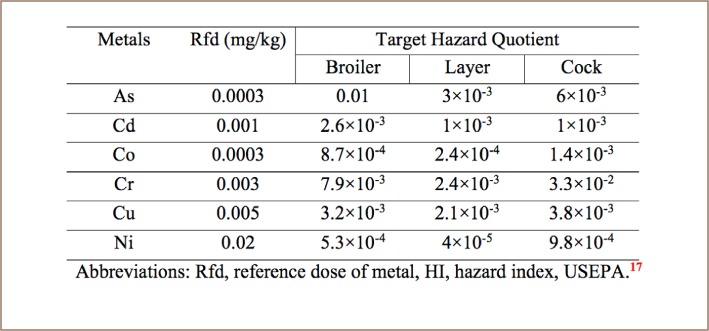
Target Hazard Quotient of Trace Metals from the Consumption of Different Types of Chicken

**Table 9:**
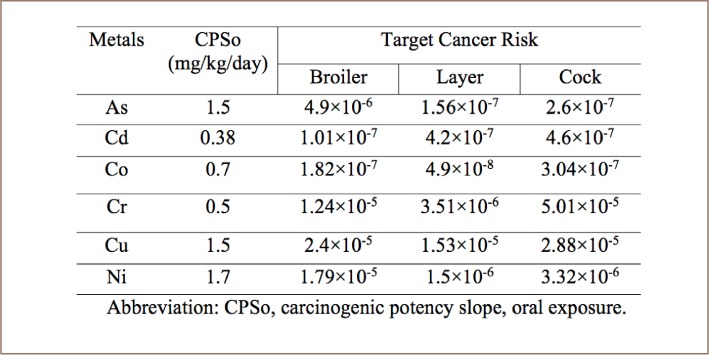
Target Cancer Risk of Trace Metals from the Consumption of Different Types of Chicken

Deposition of trace metals in chicken may be caused by contaminated feed, raw materials of feed, medicine, water and other environmental parameters. One limitation of the present study was that the deposition of trace metals was not analyzed. In addition, the population of Bangladesh is predominantly vegetarian, and other non-meat products should be tested.

## Conclusions

Trace metal concentrations in different types and parts of chickens available to consumers in various markets in Dhaka were analyzed. The results indicated that the concentrations of As, Cr, Cu in the analyzed samples exceeded the maximum permissible level, although the concentrations of Cd and Ni found were within the acceptable limits of the WHO. The high concentrations of As, Cr and Cu in some parts of chicken may be due to the metals inadvertently entering the food chain. Additional studies are needed to identify the specific source of these metals. Estimated levels of As, Cr and Cu were higher than the maximum permissible levels and indicate a health risk. Groundwater in many parts of Bangladesh is contaminated with As and may be a source of As contamination in poultry. High concentrations of Cr and Cu in poultry may be caused by contaminated poultry feed. Among the three types of investigated poultry, layers appear to be comparatively less contaminated with metals than other types of chicken. The target hazard quotient and target cancer risk values of the analyzed chicken samples were less than the acceptable limits for all individual trace metals, indicating no non-carcinogenic and cancer health risk from ingestion of a single trace metal via consumption of chicken. The authors recommend the creation of a metals monitoring program run by the Ministry of Food or Ministry of Agriculture of Bangladesh to keep poultry meat safe for human consumption. In addition, measures are needed to monitor and maintain the quality of poultry feed through periodic surveillance.
